# Pressurized Hot Water Extraction and Capillary Electrophoresis for Green and Fast Analysis of Useful Metabolites in Plants

**DOI:** 10.3390/molecules24132349

**Published:** 2019-06-26

**Authors:** Kurt Debruille, Jason A. Smith, Joselito P. Quirino

**Affiliations:** 1 Australian Centre for Research on Separation Science (ACROSS), School of Natural Sciences-Chemistry, University of Tasmania, Private Bag 75, Hobart, 7001 Tasmania, Australia; 2 Department of Chemistry, Faculty of Science, University of Mons, 20 Place du Parc, 7000 Mons, Belgium; 3 School of Natural Sciences-Chemistry, University of Tasmania, Private Bag 75, Hobart, 7001 Tasmania, Australia

**Keywords:** plant metabolites, pressurized hot water extraction, capillary electrophoresis, polygodial, coumarin, shikimic acid

## Abstract

The search for useful compounds from plants is an important research area. Traditional screening that involves isolation and identification/quantitation is tedious, time consuming, and generates a significant amount of chemical waste. Here, we present a simple, fast, and green strategy to assess ≥0.1% wt/wt quantities of useful compounds in plants/spices using pressurized hot water extraction using a household espresso machine followed by chemical analysis using capillary electrophoresis. Three demonstrations with polygodial, cinnamaldehyde, coumarin, and shikimic acid as target metabolites are shown. Direct analysis of extracts was by the developed micellar electrokinetic chromatography and capillary zone electrophoresis methods. The approach, which can be implemented in less developed countries, can process many samples within a day, much faster than traditional techniques that would normally take at least a day. Finally, 0.8–1.1% wt/wt levels of shikimic acid were found in Tasmanian-pepperberry and Tasmanian-fuschia leaves via the approach.

## 1. Introduction

Many useful compounds that benefit society are sourced from plants [1,2]. They are plant metabolites such as coumarin [3], curcumin [4], morphine [5], polygodial [6,7], and shikimic acid [8]. They are often used in the food and pharmaceutical industries, e.g., for their flavour, medicinal and biological properties. The screening of these compounds from new plant sources normally requires their isolation, which is performed using a battery of traditional techniques such as solvent extraction, crystallization, and chromatography [9,10]. These techniques can be lengthy, tedious, costly, and generate a significant amount of chemical waste. After an ample amount of purified sample is recovered, identification is by using advanced instrumental techniques including nuclear magnetic resonance (NMR), mass spectrometry (MS), and gas chromatography (GC) and liquid chromatography (LC) with known standards. Analysis of targeted metabolites in plant extracts can however be directly performed using NMR techniques [11,12,13], but NMR instrumentation is not easily accessible, especially in developing countries.

Global research on the extraction of plant metabolites using green approaches is on the rise [14,15,16,17,18]. These approaches are mainly the implementation of sustainable and environmentally-benign extraction systems, such as ionic liquids [14], deep eutectic solvents [15,16,17], and vegetable oil [18]. The extraction with green solvent systems has also been improved with the use of an external driving force such as ultrasound, microwave, temperature, and pressure [19,20,21,22]. In the early 2000s, pressurized hot water extraction (PHWE) was shown to be a green and suitable method for plant metabolites [23,24]. More recently, faster PHWE has been obtained with an unmodified espresso machine [2,7,25], with isolation of metabolites in the >1% wt/wt scale. Less chlorophylls are extracted, and therefore, cleaner extracts result. The interest in espresso machine-based PHWE has also been extended to the assay of bio-actives in other plant-based samples in conjunction with LC [26,27] and GC [28,29], where analysis was typically after sample treatment of PHWE extracts. However, the potential exists for immediate analysis of the PHWE extract to assess the potential abundance of useful metabolites. 

Capillary electrophoresis (CE) is a family of analytical techniques that uses an electric field for separation. The popular CE modes in natural products’ analysis are capillary zone electrophoresis (CZE) and micellar electrokinetic chromatography (MEKC), which separates plant metabolites by differences in electrophoretic mobility and interaction with micelles, respectively [30]. MEKC allows separation of neutral analytes, which is reminiscent of reversed phase (RP)-LC [31]. The small scales in which CE is performed makes it a greener alternative to LC [32]. For example, an RP-LC unit can generate >1 L of chemical waste (mobile phase with organic solvents) in a day, while CE uses <10 mL of separation or background solution (BGS) that is typically void of organic solvents. Another advantage of CE is that it allows the analysis of plant extracts that contain high levels of targeted metabolites without the need for tedious and multi-step sample preparation, improving the sample throughput and eliminating the excessive use of organic solvents [33,34]. 

Here, we report a green, simple, and fast approach for the screening of known useful compounds or metabolites from dried plants and spices using espresso machine-based PHWE followed by CE analysis. To show a broad applicability, three demonstrations were conducted with the targeted compounds (1) polygodial, (2) cinnamaldehyde, (3) coumarin, and (4) shikimic acid, which are present in mg/g amounts in the model plant samples *Tasmania lanceolata* (Tasmanian-mountain pepperberry) (1), *Cinnamomum cassia* (Chinese cinnamon) (2 and 3), and *Illicium verum* (star anise) (4). MEKC methods were developed for the neutral analytes (1, 2, and 3) and a CZE method for the anionic (4) analyte. CE provided sufficient separation even in the presence of sample matrix constituents, where the sample was simply diluted in water or CE BGS. The CE methods were used to study the effects of ethanol (EtOH) content and/or pH of the extraction solutions used in PHWE. Finally, PHWE and CZE were used to screen shikimic acid from different Tasmanian plants including *Tasmannia lanceolata* and *Correa backhouseana* (Tasmanian-fuchsia). 

## 2. Results and Discussion

### 2.1. Polygodial from Tasmannia Lanceolata Leaf

**Figure 1:**
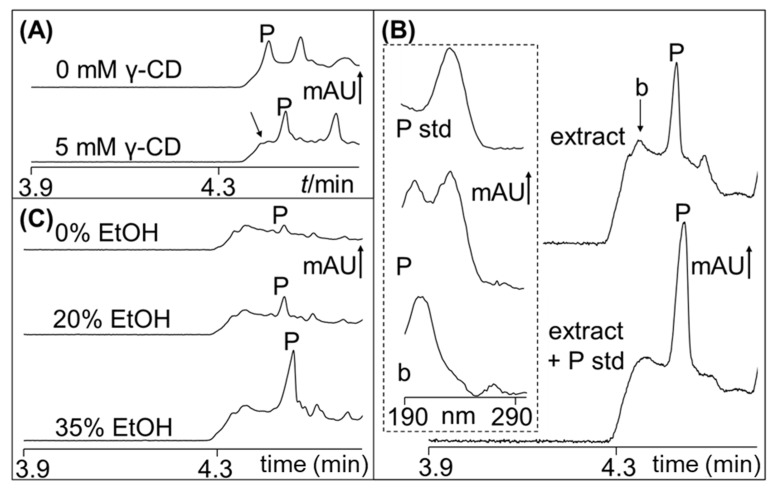
Representative micellar electrokinetic chromatography (MEKC) electrochromatograms of *Tasmannia lanceolata* leaf extracts. P is the peak of polygodial. (**A**) Effect of γ-cyclodextrin (γ-CD) addition to the background solution (BGS). BGS was 50 mM SDS, 25 mM phosphoric acid (pH 2), with 0 or 5 mM γ-CD. The sample was extracted by pressurized hot water extraction (PHWE) with 30% EtOH. (**B**) Standard addition for polygodial identification. The sample extracted with 30% EtOH was unspiked (top) or spiked (bottom) with 100 µg/mL of polygodial. The inset shows the UV spectrum of the background (b) and P (peak of polygodial with b). The spectrum on the top of the inset is from MEKC of polygodial standard (P std). (**C**) Effect of different % EtOH in the extraction solution used in PHWE. Other conditions are in the Materials and Methods Section. mAU is milli-absorbance units.

Polygodial is a neutral and hydrophobic sesquiterpene dialdehyde that is believed to be responsible for the pungent taste of several plants, including *Tasmannia lanceolata*. For fast MEKC analysis of polygodial, MEKC with SDS micelles in an acidic buffer were used [31,35]. After injection of the sample and application of voltage (anode at detector), the SDS micelles transported the analyte (i.e., polygodial with high affinity to the micelles) to the detector. The electrophoretic velocity of SDS micelles (the pseudostationary phase) was faster than the electroosmotic flow (EOF). Representative MEKC with SDS analysis of a first 60-mL PHWE extract using 30% EtOH of Tasmannia *lanceolata* leaf (15 g) is shown in Figure 1A. A shift in the baseline at around 4.4 min prevented the proper integration of the polygodial peak (P). This was solved by adding 5 mM γ-cyclodextrin (γ-CD) to the BGS, which increased the migration time of polygodial due to its interaction with γ-CD (see Figure 1A, without (top) and with (bottom) γ-CD). In the MEKC conditions in Figure 1A, SDS was electrophoretically moving to the anode, while the γ-CD was moving to the cathode due to the EOF. The analyte was brought to the detector by the faster moving SDS micelles (compared to the EOF). Identification of the polygodial peak was by spiking the extract with an authenticated standard and peak UV-spectra comparison, as shown in Figure 1B. The baseline (b) shift, which extended up to ~5 min, had a characteristic spectrum. This shift could be from non-polar compounds due to their significant affinity to the SDS micelles, as evidenced by its early elution. 

**Figure 2:**
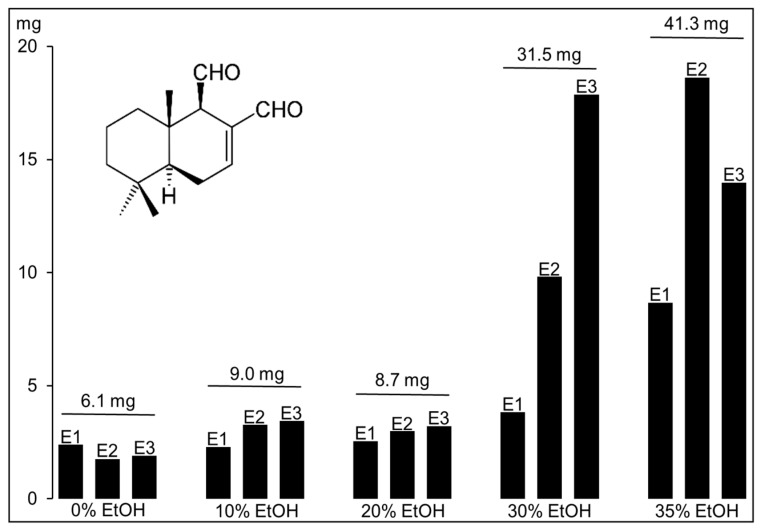
Amount of polygodial (mg) from the first (E1), second (E2), and third (E3) extracts obtained by PHWE of 15 g (each) *Tasmannia lanceolata* leaf. The total amount extracted (E1 + E2 + E3) from three consecutive PHWE is at the top of each extraction solution. Analysis was by MEKC, as described in the Materials and Methods Section and Figure 1.

The dried leaves were extracted with 0–35% EtOH, and the representative MEKC results using 0, 20, and 35% EtOH are shown in Figure 1C. There was an obvious increase in the amount of polygodial extracted (increase in the peak signals) with increasing concentration of EtOH. The samples were also extracted 3× (60-mL fractions), and the amounts of polygodial extracted in the three extracts (labelled as E1, E2, and E3) are shown in Figure 2. With 0, 10, and 20% EtOH, the total amount of polygodial extracted (sum of the amounts from the three extractions) was between 6.1 and 9.0 mg (0.04 and 0.06% wt/wt). With 30 and 35% EtOH, the total amount was significantly higher at 31.5 and 41.3 mg (0.21 and 0.28% wt/wt), respectively. Interestingly, the second 60-mL PHWE extract (E2) using 30 and 35% EtOH was able to extract approximately 2× more polygodial when compared to the corresponding first 60-mL PHWE extract (E1). The cellular disruption during the first PHWE perhaps made the second PHWE of polygodial more successful. The unknown compound (baseline shift in Figure 1) in the plant extract could have also interfered with the extraction of polygodial during the first PHWE procedure. Supplementary Information (SI) Figure S1 shows the decrease in the baseline shift intensity for the second (E2) and third (E3) PHWE using 30 and 35% EtOH. There was a concurrent increase in the peak height for polygodial in E2 and E3 when compared with the corresponding E1 (first PHWE). The improved extraction of polygodial after the first PHWE was not observed with extractions using 0–20% EtOH. The PHWE (E1, E2, and E3) with 20% EtOH is shown in SI Figure S1. The extraction behavior with different % EtOH by PHWE can be used in latter studies for more efficient extraction and recovery of polygodial. 

The previously-reported recovery of polygodial from *Tasmannia lanceolata* with PHWE and 35% EtOH was 0.33% wt/wt of dried leaf sample of a varietal with high polygodial content [7]. This value was obtained after isolation, which required liquid/liquid extraction with dichloromethane and flash chromatography. The 0.28% wt/wt (41.3 mg polygodial from E1, E2, and E3 in 15 g of sample; see Supplementary Information Figure S2) estimated by MEKC of the 35% EtOH extracts from PHWE was close to the above-reported value, although the sample batch of *Tasmannia lanceolata* processed and volumes of 35% EtOH used were not the same.

### 2.2. Cinnamaldehyde and Coumarin from Cinnamomum cassia

**Figure 3:**
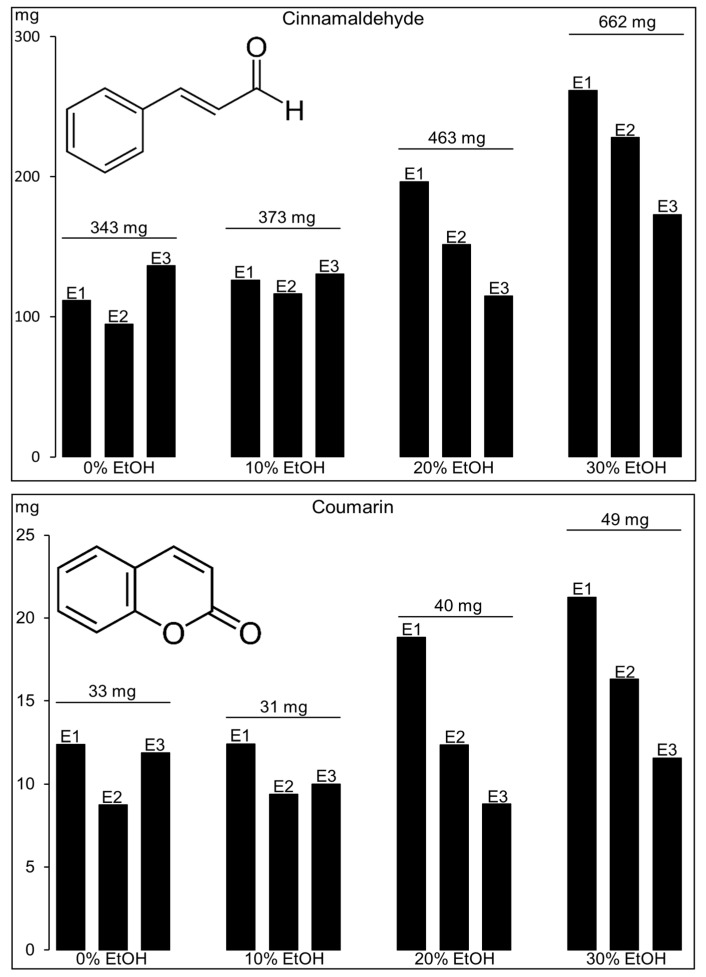
Amount (mg) of cinnamaldehyde and coumarin from the first (E1), second (E2), and third (E3) extracts obtained by PHWE of 20 g (each) of *Cinnamomum cassia* bark. The total amount extracted (E1 + E2 + E3) from three consecutive PHWE is at the top of each extraction solution. Analysis was by MEKC, as described in the Materials and Methods Section.

**Figure 4:**
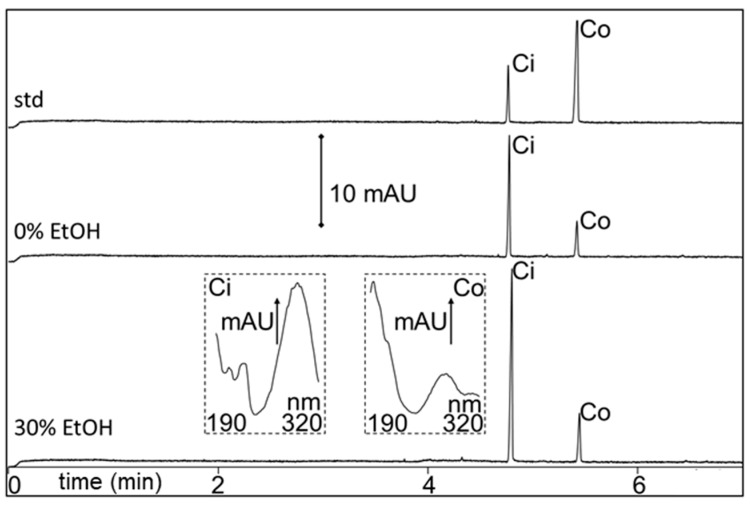
Representative MEKC electrochromatograms of (50 μg/mL each) cinnamaldehyde and coumarin standard mixture (std) and the first 0 and 30% EtOH extracts of *Cinnamomum cassia* bark sample after PHWE. Peak identity: cinnamaldehyde (Ci) and coumarin (Co). Insets show the UV spectra of the targeted metabolites. Extracts were diluted with water (1:20) before injection. Other conditions are in the Materials and Methods Section.

The applicability of PHWE and MEKC was extended to other neutral metabolites, i.e., cinnamaldehyde and coumarin, which are major plant metabolites in *Cinnamomum cassia* (mainly in the bark). Similar to the neutral polygodial, fast MEKC was performed using SDS with an acidic buffer. The addition of γ-CD was not needed, since the targeted cinnamaldehyde and coumarin separated nicely between 4 and 6 min with no significant baseline shifts produced from the sample matrix. The sample was extracted 3× by PHWE with 0–30% EtOH (three 60-mL collections). The amounts (determined by MEKC) of the targeted metabolites found are summarized in Figure 3. Representative MEKC analysis of the first 0 and 30% EtOH extracts (E1, diluted with water) are shown in Figure 4. The identity of cinnamaldehyde (Ci) and coumarin (Co) in the extracts was performed by comparison of the retention time and UV spectra from the analysis of the standards, as shown in Figure 4. The results in Figure 3 and Figure 4 clearly indicated that there was better extraction using higher % EtOH, with 30% EtOH providing the highest recovery for the metabolites. This was more pronounced with the extraction of the less polar cinnamaldehyde, as expected. The amount of cinnamaldehyde and coumarin estimated from the three-times 60-mL consecutive PHWE using 30% EtOH was 3.31% (662 mg of cinnamaldehyde from E1, E2, and E3 in 20 g of the sample; see Figure 3) and 0.25% wt/wt (49 mg coumarin from E1, E2, and E3 in 20 g of the sample; see Figure 3), respectively. Ultrasonic extraction with 50% methanol for 30 min of powdered *Cinnamomum cassia* bark followed by LC-MS analysis (with ACN in the mobile phase) revealed comparable values of 2.61 and 0.34% wt/wt for cinnamaldehyde and coumarin, respectively [36]. 

### 2.3. Shikimic Acid from Illicium verum and Other Plants

**Figure 5:**
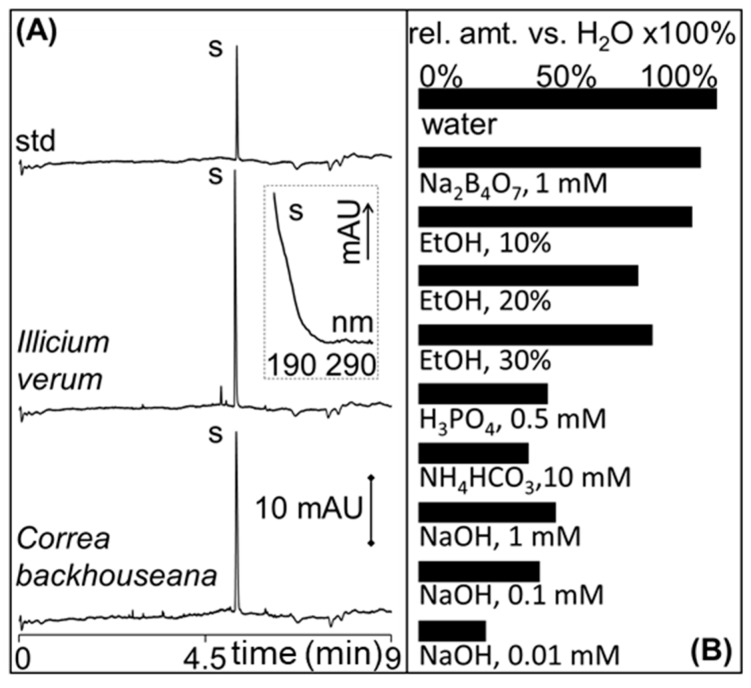
(**A**) Representative capillary zone electrophoresis (CZE) electropherograms of (50 μg/mL) shikimic acid standard (std) and the first PHWE extract with water of *Illicium verum* (1:50 dilution) and *Correa backhouseana* (1:20 dilution). The extracts were diluted with water. S is the peak for shikimic acid, and its UV spectrum is shown in the inset. Other conditions are in the Materials and Methods Section. (**B**) Shows the relative amount of shikimic acid found using different extraction solutions versus the amount found using water in PHWE × 100%. Calculations were based on the first 60 mL PHWE extract.

Shikimic acid from *Illicium verum* is used as a precursor for the synthesis of oseltamivir [37]^,^ [38]. The pKa of shikimic acid is 4.1; thus, shikimic acid was analyzed as a negatively-charged analyte by CZE using ammonium bicarbonate buffer at pH 9.5. Representative CZE analysis of shikimic acid standard solution and the first PHWE extract using water as the extraction solvent of *Illicium verum* are shown in Figure 5A. The identity of shikimic acid in the extracts was by comparison of the retention time and UV spectra (see the inset in Figure 5A) from the analysis of the standard. CZE was used to assess rapidly the extraction efficiency of different extraction solutions vs. water in PHWE, and the results are shown in Figure 5B. The relative amount of shikimic acid (rel. amt. vs. H_2_O × 100%) was calculated by dividing the amount of shikimic acid from each 60-mL extract (different % EtOH, concentrations of NaOH, and type of buffer) by the amount obtained with water multiplied by 100%. PHWE with water provided the best extraction efficiency, while the addition of EtOH reduced the extraction efficiency (rel. amt. vs. H_2_O < 100%). The amount of shikimic acid determined by CZE from the first 60-mL PHWE using water was 1.6% wt/wt. The amount of shikimic acid in the second and third PHWE decreased to 1.3 and 1.0% wt/wt, respectively. After the sixth PHWE, the shikimic acid was almost exhausted from the sample. The amount found in the first three extractions (3.9% wt/wt) (780 mg of shikimic acid in 20 g of sample) was lower than the previously-reported value with PHWE using 30% EtOH of 5.5% wt/wt [25]. The *Illicium verum* sample processed in this study was however from a different source and in storage at room temperature for many years. We also note that higher values of shikimic acid can be found in other *Illicium verum* samples, e.g., up to ~8% [39]. On a different note, the use of basic solutions has been shown to improve the extraction of shikimic acid in *Illicium verum* [40]. However, our results (with alkaline solutions of NaOH and NH_4_HCO_3_) showed that this is not applicable in PHWE of shikimic acid (rel. amt. vs. H_2_O << 100%). 

**Figure 6:**
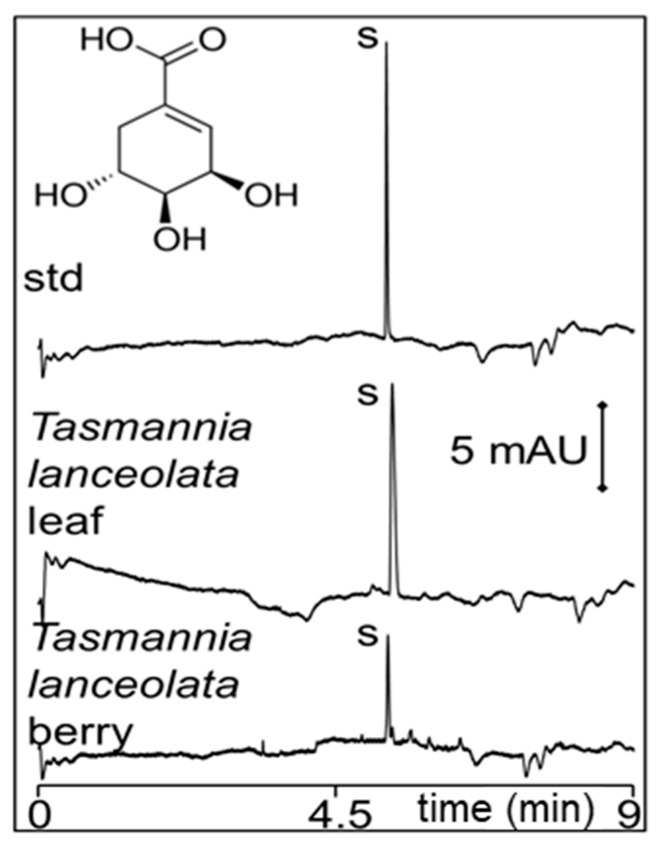
Representative CZE electropherograms of (50 μg/mL) shikimic acid in-house standard (std) and the first PHWE extract with water of *Tasmannia lanceolata* leaf (1:20 dilution) and berry (1:4 dilution). The extracts were diluted with water. Other conditions are in the Materials and Methods Section.

PHWE with water and CZE was then used for the screening of shikimic acid in several plants including *Tasmannia lanceolata* (leaf and berry), *Backhousia citriodora*, *Glycyrrhiza glabra*, *Correa backhouseana*, and *Dodonaea viscosa*. From the first 60-mL PHWE, the amount of shikimic acid found in *Correa backhouseana* and *Tasmannia lanceolata* leaf was significant at 1.1 and 0.8% wt/wt, respectively. A small amount of 0.01% wt/wt was found in *Tasmannia lanceolata* berries. Representative CZE analysis of *Correa backhouseana* extract is shown in Figure 5A, while those for *Tasmannia lanceolata* leaf and berry are in Figure 6.

## 3. Materials and Methods 

### 3.1. Reagents, Standards, and Solutions

Reagents (acetonitrile (ACN), ammonium bicarbonate (NH_4_HCO_3_), EtOH, γ-CD, methanol (MeOH), phosphoric acid (H_3_PO_4_), poly(allyldimethylammonium chloride (PDDAC) with an average molecular weight of 400–500k, poly(styrene) sulfonate (PSS) with an average molecular weight of 70k, sodium hydroxide (NaOH), sodium dodecyl sulfate (SDS), and sodium tetraborate (Na_2_B_4_O_7_)) were obtained from Sigma-Aldrich (St. Louis, MO, USA). Purified water was from a Milli-Q system (Millipore, MA, USA). The pH of the solutions was measured using a bench top meter (Sper Scientific, Australia). 

Stock solutions (for BGS preparation) including 0.5 M NH_4_HCO_3_ (pH 9.5), 1 M H_3_PO_4_ (pH 2), and 0.2 M SDS were sonicated and filtered using a 0.45-µm filter prior to use. MEKC BGS for polygodial was prepared by mixing appropriate volumes of H_3_PO_4_ and SDS stock solutions and a weighed amount of γ-CD with purified water. MEKC BGS for cinnamaldehyde and coumarin was prepared by mixing appropriate volumes of H_3_PO_4_ and SDS stock solutions with purified water. CZE BGS for shikimic acid was prepared by mixing an appropriate volume of NH_4_HCO_3_ stock solution with purified water. The polyelectrolyte solutions used for coating the capillary in CZE were 1% PDDAC and 1% PSS in purified water. 

The extraction solutions (0, 10, 20, 30, and 35% EtOH) for PHWE were prepared by mixing appropriate volumes of EtOH and purified water. Additional extraction solutions used for shikimic acid were prepared by mixing appropriate volumes of H_3_PO_4_, NH_4_HCO_3_, and Na_2_B_4_O_7_ with purified water. The 100 mM stock solution of Na_2_B_4_O_7_ was prepared with purified water. 

Standards of cinnamaldehyde and coumarin were purchased from Sigma-Aldrich. Shikimic acid and polygodial standards were isolated by literature methods [7,25]. Standard stock solutions (1 mg/mL) were prepared in ACN (polygodial), 10% MeOH (coumarin), and 50% MeOH (shikimic acid and cinnamaldehyde) and stored at 4–8 °C when not in use. Sample solutions were prepared by mixing appropriate amounts of analyte stock solutions with water or BGS. 

### 3.2. Plant Materials

*Tasmannia lanceolata* (berry and ground leaf) was purchased from Diemen Pepper, Birchs Bay, Tasmania, Australia. *Backhousia citriodora* (lemon myrtle) (leaf), *Cinnamomum cassia* (dried bark), *Glycyrrhiza glabra* (liquorice) (root), and *Illicium verum* (dried fruit and seed) were purchased from specialty stores in Hobart, Tasmania. *Correa backhouseana* (leaf) and *Dodonea viscosa* (hopbush) (leaf) were collected at the University of Tasmania, Sandy Bay campus, and then air dried. The samples (except *Tasmannia lanceolata*) were ground using a household multi-grinder prior to extraction.

### 3.3. Espresso Machine-Based PHWE

PHWE was performed with a Breville Australia Model BES820/A espresso machine. Plant material (8–20 g) was placed in the filter basket. The exact quantities of materials used are indicated in the SI Table S1. For each PHWE, samples were extracted, and 60-mL extracts were collected. Each extract was stored at 4–8 °C when not in use. An aliquot of an extract was centrifuged (1000 rpm) for 3 min, and the supernatant was passed through a 0.45-µm filter prior to further processing.

### 3.4. CE Procedures

CE was performed using an Agilent Technologies 3D-CE instrument with a diode array detector (Waldbronn, Germany). Capillary separation was performed with fused-silica from Polymicro (Phoenix, AZ, USA) with 50 μm × 360 μm (i.d. × o.d.). The temperature of the capillary cartridge was controlled at 20 °C. The spectrum during each run was collected from 190–320 nm. New capillaries were flushed at 1 bar with 0.1 M NaOH (15 min) and purified water (15 min) prior to use. Sample injection was at 25 mbar for 6 s. 

MEKC BGS for polygodial was performed with 50 mM SDS, 25 mM phosphoric acid (pH 2), and 5 mM γ-CD. Separation voltage and detection were 20 kV and 225 nm, respectively. MEKC BGS for cinnamaldehyde and coumarin was performed with 50 mM SDS and 25 mM phosphoric acid (pH 2). Voltage and detection were 20 kV and 200 nm, respectively. After each run in the MEKC methods, the capillary was conditioned with purified water (1 min), 0.1 M NaOH (1 min), purified water (2 min), and BGS (4 min). CZE BGS for shikimic acid was performed with 100 mM NH_4_HCO_3_ (pH 9.5). Separation voltage and detection were 20 kV and 200 nm, respectively. The EOF was reversed or anodic (co-EOF CZE separation) by using a successive multiple ionic layer of PSS and PDDAC to form a seven-layer coating that ended with PDDAC (positive surface wall layer). After each run, the capillary was conditioned with purified water (1 min), 1% PDDAC (0.5 min), purified water (1 min), and BGS (4 min). All separations were performed at negative polarity (anode at the inlet). Corrected peak areas were calculated by dividing the peak area with the migration (CZE) or retention (MEKC) time. The concentrations of the targeted metabolites were determined by comparison to an external standard. The extracts were analyzed at least 2×, and the averaged values are reported. The % g of targeted metabolite/s per g of dried plant sample (%wt/wt) was calculated using the concentrations obtained from CE analysis, the dilution factor, the weight of plant material, and the volume of extraction solution collected in PHWE.

### 3.5. CE Methods Selection/Optimization 

The PHWE (first 60-mL extract) using 30% EtOH (*Tasmannia lanceolata*) and water (*Cinnamomum cassia* and *Illicium verum*) was used to optimize the CE methods. The low pH BGS in the MEKC method allowed a fast elution (within 6 min) of neutral metabolites polygodial, cinnamaldehyde, and coumarin in the samples. The SDS concentration (25–100 mM tested) was optimized, with 50 mM providing the best performance. The γ-CD concentration (5–20 mM tested) in the BGS was optimized for polygodial analysis. The co-EOF CZE with high pH buffer using NH_4_HCO_3_ allowed the fast migration (within 7 min) of shikimic acid as an anionic analyte. The concentration range, linearity, and repeatability of the MEKC and CZE methods are summarized in SI Table S2. The analytical figures of merit were found suitable for screening of metabolites in plant extracts.

### 3.6. Sample Preparation after PHWE

We found that the resulting concentration of the targeted metabolites from the first three 60-mL extracts obtained from the model plants were high and outside the linear range of the CE methods. Dilution of the extracts with water or BGS (4–50 times) allowed the quantitation of the metabolites. This simple approach circumvented the tedious sample preparation or purification steps typically required in the search for plant metabolites. 

## 4. Conclusions

A fast, effective, and absolutely green screening strategy for known useful compounds in dried plants and spices based on household espresso machine-based PHWE and CE was presented. PHWE with water afforded efficient extraction with resulting concentrations of targeted metabolites at sufficient levels that could be directly analyzed after appropriate dilution by CE. For neutral metabolites, although higher recoveries were obtained with ≥30% EtOH in PHWE, the screening can be accomplished simply with water as extraction liquid. Optimization of the CE modes of CZE and MEKC for the charged and neutral metabolites, respectively, was easily realized due to the high separation power of CE for small molecules. CE was also found to be effective for the rapid assessment of the extracts from PHWE using different extraction solutions. The proposed PHWE with water and CZE with purely aqueous background electrolyte for the screening of shikimic acid in different plants was successfully implemented. The leaves of the plants *Correa backhouseana* and *Tasmannia lanceolata* were found to contain significant levels of the useful anionic metabolite. This approach has the potential to be utilized for the rapid screening of metabolites in plants and to optimize extraction protocols.

## Supplementary Materials

The following are available online.
