# Supplementary material for: Pressurized Hot Water Extraction and Capillary Electrophoresis for Green and Fast Analysis of Useful Metabolites in Plants
Source: Molecules. 2019 Jun 26;24(13):2349. doi: 10.3390/molecules24132349 (PMC6651437; doi:10.3390/molecules24132349)
Supplement: Supplementary file 1 [file molecules-24-02349-s001.pdf]

Supplementary Information (SI)

Pressurized hot water extraction and capillary electrophoresis for green and fast analysis of useful metabolites in plants

Kurt Debruille[1,2], Jason Smith[3], and Joselito P. Quirino[1,*]

[1]Australian Centre for Research on Separation Science (ACROSS), School of Natural Sciences-Chemistry, University of Tasmania, Private Bag 75, Hobart, Tasmania 7001, Australia

[2]Department of Chemistry, Faculty of Science, University of Mons, 20 Place du Parc, Mons 7000, Belgium

[3]School of Natural Sciences-Chemistry, University of Tasmania, Private Bag 75, Hobart, Tasmania 7001, Australia

* corresponding author: jquirino@utas.edu.au

Table S1. Quantities of plant sample extracted by PHWE. (page 2)

Table S2. Concentration range, linearity, and repeatability of the CE methods for the analysis of the targeted metabolites. (page 3)

Figure S1. MEKC profiles of the successive PHWE extracts 1, 2, and 3 (E1, E2, and E3, respectively) using 20, 30, and 35% EtOH of *Tasmannia lanceolata* leaf. (page 4)

Table S1. Quantities of plant sample extracted by PHWE

Sample (dried)	quantities (g ± 0.01 g)	targeted metabolite/s
Tasmannia lanceolata (leaf)	15	polygodial
Cinnamomum cassia (bark)	20	cinnamaldehyde and coumarin
Illicium verum (fruit and seed)	20	shikimic acid
Correa backhouseana (leaf)	8	shikimic acid
Tasmannia lanceolata (pepperberry)	18.3	shikimic acid
Tasmannia lanceolata (leaf)	20	shikimic acid
Backhousia citriodora (leaf)	13.4	shikimic acid
Dodonea viscosa (leaf)	8.4	shikimic acid
Glycyrrhiza glabra (root)	15	shikimic acid

Table S2. Concentration range, linearity, and repeatability of the CE methods for the analysis of the targeted metabolites.

	linear range (µg/mL)	linear equation y= ax + b	linearity (R^2) corrected peak area	LOD (µg/mL)
polygodial	10 - 250	y = 0.048x + 0.722	0.990	3
shikimic acid	5 - 100	y = 0.254x + 6.534	0.996	2
cinnamaldehyde	1 - 100	y = 0.129x - 0.012	0.997	0.3
coumarin	1 - 50	y = 0.062x + 0.142	0.998	2

	intraday %RSD (n=8)		interday %RSD (n=8)	
	migration / retention time	corrected peak area	migration / retention time	corrected peak area
polygodial	1.3	13.2	3.5	10.5
shikimic acid	0.7	8.9	0.6	8.6
cinnamaldehyde	2.3	4.2	2.3	5.6
coumarin	0.9	1.7	1.8	10.3

MEKC and CZE methods are described in the main text.

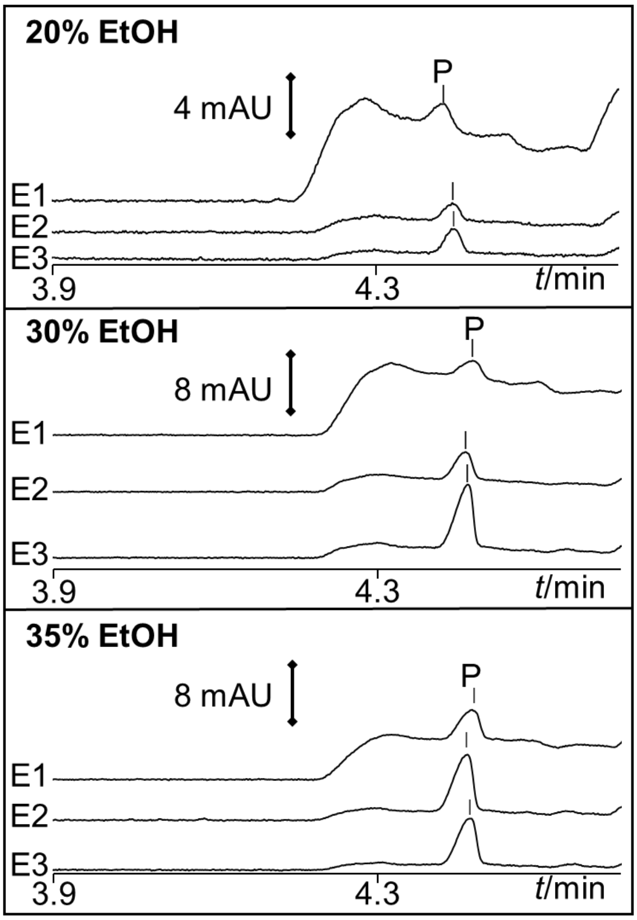

Figure S1. MEKC profiles of the successive PHWE extracts 1, 2, and 3 (E1, E2, and E3, respectively) using 20, 30, and 35% EtOH of *Tasmannia lanceolata* leaf. The intensity of the baseline shift that started <4.3 min was highest with the first extract (E1) using all extraction solutions. The intensity of polygodial peak was higher in the second and third extracts using ≥30% EtOH, indicating the improved extraction of polygodial after the first extraction using higher concentrations of EtOH. Analysis was by MEKC as described in the Materials and methods section.
